# Periodic switching of acoustic radiation force with beat created by multitone field

**DOI:** 10.1038/s41598-022-19077-9

**Published:** 2022-09-02

**Authors:** Hiroya Tanaka, Keita Funayama, Yukihiro Tadokoro

**Affiliations:** Toyota Central Research & Development Laboratories., Inc., Nagakute, 480-1192 Japan

**Keywords:** Acoustics, Fluid dynamics

## Abstract

Acoustic radiation force plays a key role in microfluidic systems for particle and cell manipulation. In this study, we investigate the acoustic radiation force resulting from synthesized ultrasounds that are emitted from multiple sound sources with slightly different oscillation frequencies. Due to the synthesized field, the acoustic radiation force is expressed as the sum of a dc component and harmonics of fundamental frequencies of a few hertz. This induces the beat of the acoustic radiation force. We demonstrate that the synthesized field provides the periodic on/off switching of the acoustic radiation force associated with the one denominational planar standing wave in a straight microfluidic channel. Consequently, our system can temporally manipulate acoustic radiation force without active controls.

## Introduction

Acoustic radiation force is a widely studied topic and continues to generate interest in fields ranging from simple dynamics^1–3^ to the broad applications such as acoustic levitators^4–6^, tweezers^7,8^, and displays^9,10^. In addition, recent developments in microfabrication technologies have enabled the integration of ultrasound transducers in the microfluidic systems, which has led to further interest in acoustic radiation force^11^. Acoustic radiation force realizes contact-free particle and cell manipulations such as concentration^12,13^, trapping^14,15^, and separation^16–18^, based on their acoustomechanical properties.

Acoustic radiation force is induced by the scattering of the acoustic waves on a small particle^19–22^. The observed motion of the small particle is not resolved on the time scale of kHz/MHz ultrasound waves^23,24^. Consequently, acoustophoretic motion is generated by the force averaged over the oscillation cycle. When the monotone acoustic wave is incident on the small particle, the scattering of acoustic waves gives rise to the time-invariant force in steady microfluidic channels.

However, recent studies have analyzed the time dependency of acoustophoretic motion. For example, the oscillating sharp-edge structure attracts or repels the particles in the microfluidic channel over a period of several seconds^25^. Meanwhile, it has been demonstrated that short acoustic pulses can increase the acoustic trapping selectivity^26,27^. Two denominational patterns of the acoustic radiation force are obtained by tuning the transient acoustic fields. Furthermore, experiments have shown that two mutually interfering acoustic fields yield amplitude modulation over time^28^. A slight frequency difference between the acoustic fields leads to a slow phase shift and causes a local rotation of the nodal pressure for the particle clumps in the fluidic chamber.

In this study, we theoretically analyze the time dependency of the acoustic radiation force at a slow time scale of several seconds when multiple ultrasounds are simultaneously incident on a small spherical particle. The synthesized ultrasounds are emitted from multiple sound sources with marginally different frequencies. To capture the dynamics, we separate the time scale into fast ($\sim$ time period of ultrasound oscillation) and slow ($\sim$ a few seconds). Subsequently, the acoustic radiation force at the slow time scale is expressed as the sum of the time-invariant component and the harmonics of the fundamental angular frequency of a few Hz. This indicates that the response of the acoustic radiation force in time can be controlled with multiple sources without active controls. To provide an instance of this control, we experimentally demonstrate that the synthesized field allows for the periodic on/off switching of the acoustic radiation force for one denominational planar standing wave in the straight microfluidic channel. In addition, we analyze the tunability of time response in terms of the amplitude of the harmonics.

Typically, studies have discussed the acoustic radiation force in the monotone field oscillating with the specific frequency. The monotone field induces a time-invariant acoustic radiation force, unlike the multitone field that induces a fluctuating or time-variant radiation force. This time-variant force comes from down-converted harmonic series, which serves as the beat of the acoustic radiation force. We rigorously analyze the beat of the acoustic radiation force based on the general theoretical model. Moreover, our approach reveals the methodology for the manipulation of the acoustic radiation force over a period without active controls.

For the active control over time, the acoustic radiation force should be adaptively strengthened by tuning the voltage applied to the sound transducers. Adaptive voltage control requires complex integrated circuits with surrounding components, increasing the system size. However, our system provides a method for temporal manipulation of the acoustic radiation force without active control and complex circuity. Thus, our manipulation methodology reduces the system sizes in programmable microfluidic channels.

## Results

### The model

**Figure 1:**
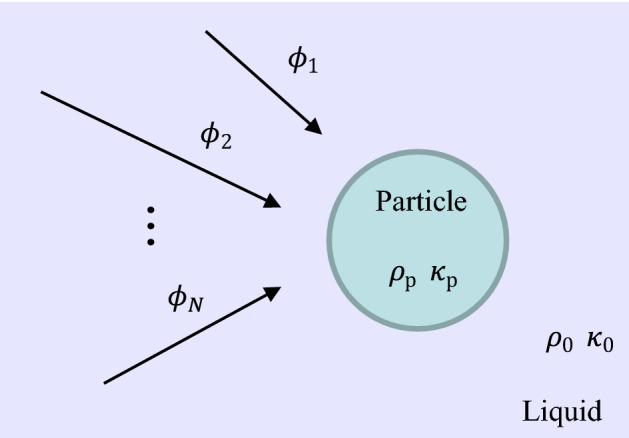
Schematic of the analytical model. Multiple incoming acoustic waves with potentials, $\phi _1,\ldots ,\phi _N$, of the different angular frequency, $\omega _1,\ldots ,\omega _N$, are incident on a small compressible spherical particle of compressibility $\kappa _{\mathrm {p}}$ and density $\rho _{\mathrm {p}}$. Such acoustic waves are synthesized in space and scatter at the particle surface. The particle is surrounded by the compressible inviscid bulk fluid of compressibility $\kappa _0$ and density $\rho _0$.

We focus on a spherical particle in the microfluidic channels and analyze the acoustic radiation force resulting from the scattering of the acoustic waves by the particle. Figure 1 illustrates the concept of our analysis. We impose an ultrasound field on the fluid containing small particles that are significantly smaller than the ultrasound wavelength. Assuming that the fluid is inviscid and incomprehensive, we consider the fluid motion in linear approximation. The corresponding Navier–Stokes equations are

1 $$\begin{aligned} \frac{\partial \rho }{\partial t} + \rho _0 \varvec{\nabla } \cdot \varvec{v}&= 0, \end{aligned}$$

2 $$\begin{aligned} \frac{\partial \varvec{v}}{\partial t} + \frac{1}{\rho _0} \varvec{\nabla } p&= 0, \end{aligned}$$

where $\rho$ is the fluid density, $\rho _0$ is the equilibrium fluid density, $\varvec{v}$ is the fluid velocity, $p=c_0^2 \rho$ is the fluid pressure, and $c_0$ is the speed on the sound in the fluid.

According to Gor’kov’s theory^3^, the acoustic radiation force acting on the small particle is expressed as

3 $$\begin{aligned} \varvec{F}(\varvec{r},t)&= -\frac{4\pi }{3}a^3\varvec{\nabla } \left[ \frac{1}{2}\mathrm {Re}[f_1] \kappa _0 \left\langle p^2 \right\rangle -\frac{3}{4}\mathrm {Re}[f_2] \rho _0 \left\langle v^2 \right\rangle \right] , \end{aligned}$$

where *a* is the particle radius, $f_1=1-\kappa _{\mathrm {p}}/\kappa _{0}$, $f_2=2(\rho _{\mathrm {p}}-\rho _0)/(2\rho _{\mathrm {p}}+\rho _0)$, $\kappa _0$ (or $\kappa _{\mathrm {p}}$) is the comprehensibility of the fluid (or particle), $\rho _{\mathrm {p}}$ is the equilibrium density of the particle, $v=|\varvec{v}|$ is the absolute value of the velocity, and the notation $\mathrm {Re}[\cdot ]$ is the real part of the complex variable. The notation $\left\langle x \right\rangle$ is the time average over an oscillation period $\tau$:

4 $$\begin{aligned} \left\langle x \right\rangle&\equiv \frac{1}{\tau } \int _0^\tau x \mathrm {d}t. \end{aligned}$$

Introducing the velocity potential $\phi$, the pressure and velocity are calculated by $p = -\rho _0 (\partial \phi /\partial t)$ and $\varvec{v} = \varvec{\nabla } \phi$. It should be noted that the force $\varvec{F}(\varvec{r},t)$ is a function of the spatial coordinate $\varvec{r}$ and time *t*.

Here, we introduce incident acoustic waves from multiple sound sources, see Fig. 1. Then, we have the time-varying multitone field:

5 $$\begin{aligned} \phi = \sum _{n=1}^{N} \phi _{n} \cos (\omega _n t +\theta _n), \end{aligned}$$

with amplitude $\{\phi _1,\ldots ,\phi _N\}$, angular frequency $\{\omega _1,\ldots ,\omega _N\}$, and relative phase $\{\theta _1,\ldots ,\theta _N\}$. The pressure and velocity are expressed as

6 $$\begin{aligned} p&= \sum _{n=1}^{N} p_{n} \sin (\omega _n t +\theta _n), \end{aligned}$$

7 $$\begin{aligned} \varvec{v}&= \sum _{n=1}^{N} \varvec{v}_{n} \cos (\omega _n t +\theta _n), \end{aligned}$$

where $$p_{n}=\rho _0 \phi _n \omega _n$$ and $$\varvec{v}_n = \varvec{\nabla } \phi _n$$.

From Eqs. (6) and (7), the time average of the squared-pressure and velocity are written as

8 $$\begin{aligned} \left\langle p^2 \right\rangle =&\left\langle \sum _{m=1}^{M} \sum _{n=1}^{N} \left( -\frac{p_{m} p_{n}}{2} \right) \cos [(\omega _m+\omega _n) t + \theta _m+\theta _n] + \sum _{m=1}^{M} \sum _{n=1}^{N} \frac{p_{m} p_{n}}{2} \cos [(\omega _m-\omega _n) t + \theta _m-\theta _n] \right\rangle , \end{aligned}$$

9 $$\begin{aligned} \left\langle v^2 \right\rangle =&\left\langle \sum _{m=1}^{M} \sum _{n=1}^{N} \frac{\varvec{v}_{m} \cdot \varvec{v}_{n}}{2} \cos [(\omega _m+\omega _n) t + \theta _m+\theta _n] + \sum _{m=1}^{M} \sum _{n=1}^{N} \frac{\varvec{v}_{m} \cdot \varvec{v}_{n}}{2} \cos [(\omega _m-\omega _n) t + \theta _m - \theta _n] \right\rangle . \end{aligned}$$

We now assume a marginal frequency discrepancy in the synthesized fields, i.e., $\omega _n = \omega _0+(n-1)\delta \omega$ and $\delta \omega \ll \omega _0$. Thus, we can distribute the time scale between the dynamics of the fast and slow time scales in Eqs. (8) and (9). The first terms describing the fast oscillation become zero in the time average operation because the acoustophoretic motion does not have the resolution on the millisecond/microsecond time scale of kHz/MHz ultrasound waves. More specifically, we have $\left\langle \cos [\{2\omega _0+(m+n-2) \delta \omega \} t + \theta _m+\theta _n]\right\rangle =0$. In contrast, the second terms take nonzero value in the time average since it varies in slow time scale compared to the oscillation cycle, i.e., $2\pi/\delta \omega \gg \tau$. Therefore, we can rewrite Eqs. (8) and (9) as

10 $$\begin{aligned} \left\langle p^2 \right\rangle&= \sum _{m=1}^{M} \sum _{n=1}^{N} \frac{p_{m} p_{n}}{2} \cos [(m-n) \delta \omega t + \theta _m-\theta _n] , \end{aligned}$$

11 $$\begin{aligned} \left\langle v^2 \right\rangle&= \sum _{m=1}^{M} \sum _{n=1}^{N} \frac{\varvec{v}_{m} \cdot \varvec{v}_{n}}{2} \cos [(m-n) \delta \omega t + \theta _m-\theta _n]. \end{aligned}$$

When we assume the quantized relative phase $\theta _n-\theta _m\in [0,\pi ]$, we can introduce the sign function, $s_{j, i+j}$. Substituting Eqs. (10) and (11) to Eq. (3), we have

12 $$\begin{aligned} \varvec{F}(\varvec{r},t)&= \sum _{i=0}^{N-1} \varvec{F}_i(\varvec{r}) \cos (i\delta \omega t), \end{aligned}$$

where

13 $$\begin{aligned} \varvec{F}_i(\varvec{r})&= -\frac{4\pi }{3} a^{3} \varvec{\nabla } \left[ \frac{1}{2}\mathrm {Re}[f_1] \kappa _0 \frac{f_i^{(\mathrm {p})}}{2} -\frac{3}{4}\mathrm {Re}[f_2] \rho _0 \frac{f_i^{(\mathrm {v})}}{2} \right] , \end{aligned}$$

14 $$\begin{aligned} f_i^{(\mathrm {p})}&= \sum _{j=1}^{N-i} s_{j, i+j} \alpha _i p_j p_{i+j} , \end{aligned}$$

15 $$\begin{aligned} f_i^{(\mathrm {v})}&= \sum _{j=1}^{N-i} s_{j, i+j} \alpha _i \varvec{v}_j \cdot \varvec{v}_{i+j} , \end{aligned}$$

16 $$\begin{aligned} s_{j, i+j}&= \left\{ \begin{array}{ll} 1 &{} \hbox { if}\ \theta _j-\theta _{i+j}=0 \\ -1 &{} \hbox { if}\ \theta _j-\theta _{i+j}=\pm \pi \end{array} \right. , \end{aligned}$$

17 $$\begin{aligned} \alpha _i&= \left\{ \begin{array}{ll} 1 &{} \hbox { if}\ i=0 \\ 2 &{} \hbox { if}\ i\ge 1 \end{array} \right. . \end{aligned}$$

It should be noted that the function $\varvec{F}_i(\varvec{r})$ specifies the spatial distributions of the magnitude of the acoustic radiation force. In addition, this distribution varies in time resulting from the harmonics $\cos (i\delta \omega t)$ at the fundamental angular frequency $\delta \omega$.

### Periodic switching of acoustic radiation force created by one dimensional standing wave

The time response of the acoustic radiation force can be designed according to the amplitudes and relative phases of the incident ultrasounds. Here, we theoretically demonstrate the periodic on/off switching without active controls for an instance of the design of the time response.

We now analyze one dimensional planar standing wave generated by the multitone ultrasound in the straight microfluidic channel. Considering the boundary condition at the side walls of one dimensional channel, we have a synthesized field^24^,

18 $$\begin{aligned} p&= \sum _{n=1}^{N} p_{n} \cos (k z) \sin (\omega _n t +\theta _n), \end{aligned}$$

19 $$\begin{aligned} \varvec{v}&= \begin{pmatrix} 0 \\ 0 \\ \sum _{n=1}^{N} \left( -\frac{p_n}{\rho _0 c_0}\right) \sin (k z) \cos (\omega _n t +\theta _n) \end{pmatrix}, \end{aligned}$$

where $k=2\pi /\lambda _0=\omega _0/c_0$, $\lambda _0/2=l$, and *l* is the channel width. We assume that the incident waves with each angular frequency $\omega _n$ $(n=1,\ldots , N)$ creates a similar force distribution, i.e, $\sin (2k_n z) \approx \sin (2k z)$ for $k_n = \omega _n/c_0$ at $n=1,\ldots ,N$.

Substituting Eqs. (18) and (19) to Eq. (3), we have the following relation,

20 $$\begin{aligned} F(z,t) = F_{\mathrm {z}}(z) F_{\mathrm {t}}(t), \end{aligned}$$

where

21 $$\begin{aligned} F_{\mathrm {z}}(z)&= 4\pi \Phi k a^3 E \sin (2kz), \end{aligned}$$

22 $$\begin{aligned} F_{\mathrm {t}}(t)&= \sum _{i=0}^{N-1} w_i \cos (i\delta \omega t) , \end{aligned}$$

23 $$\begin{aligned} w_i&= \sum _{j=1}^{N-i} s_{j, i+j} \alpha _i {\tilde{p}}_j {\tilde{p}}_{i+j}, \end{aligned}$$

and $\Phi = f_1 /3+f_2/2$, $E = (\sum _{n=1}^{N}p_n^2)/(4\rho _0c_0^2)$, and ${\tilde{p}}_j=p_j/\sqrt{\sum _{n=1}^{N}p_n^2}$ . Note that $\Phi$ is the the acoustophoretic contrast factor^24^, and *E* is the acoustic energy density.

**Figure 2:**
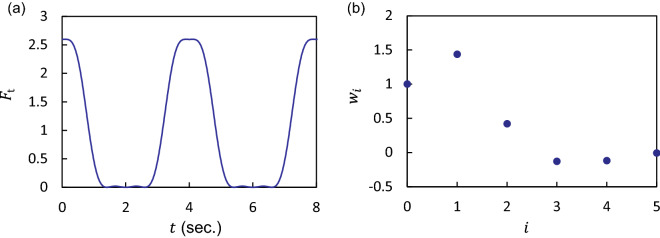
(**a**) Variation in time of $F_{\mathrm {t}}$, which is designed to be periodically switched acoustic radiation force. (**b**) Weight $w_i$, allowing for the periodic switch. We set the frequency differential to $\delta \omega/(2\pi) =$ 0.25 Hz and the number of the incident waves to $N=6$. The amplitudes are $\tilde{p}_1=0.02854$, $\tilde{p}_2=0.4314$, $\tilde{p}_3=0.72149$, $\tilde{p}_4=0.51841$, $\tilde{p}_5=0.05564$, and $\tilde{p}_6=0.14385$. The phases are $\theta _1=\cdots =\theta _5=0$ and $\theta _6=\pi$.

**Figure 3:**
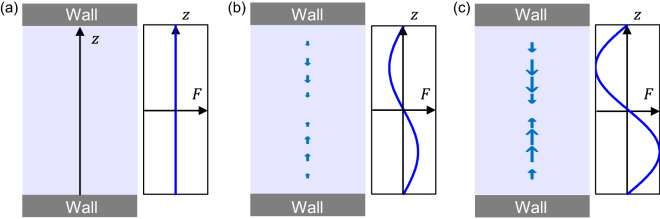
Left panels: cross-section sketches of a straight, hard-walled water-filled channel. The acoustic radiation force is illustrated by blue arrows at time (**a**) $t=2$ s, (**b**) 3.2 s, and (**c**) 4 s. Right panels: transverse and standing ultrasound wave in the channel.

**Figure 4:**
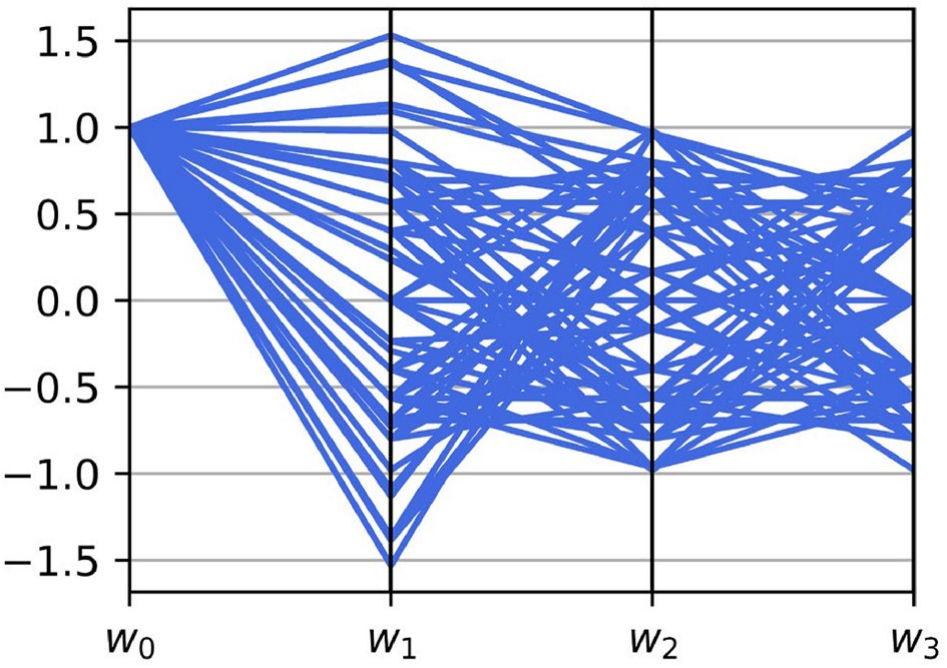
Possible values of the weight $w_i$ at $N=4$ with the constraint of $\sum _{n=1}^N \tilde{p}_n^2=1$ on parallel coordinates. The value of $\tilde{p}_i$ for each *i* is incremented by 0.2.

**Figure 5:**
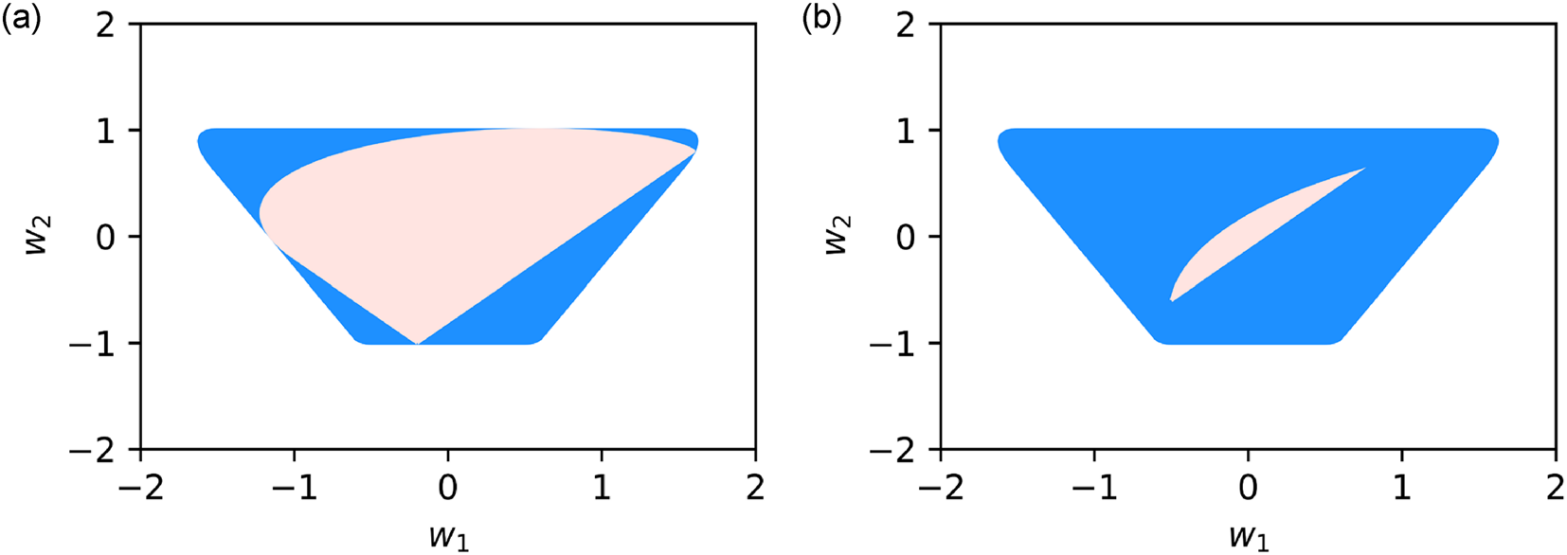
Possible range of the weights $w_1$ and $w_2$ at $N=4$ with the constraint as $\sum _{n=1}^{N} \tilde{p}_n^2 =1$ and the fixed value of $w_3$: (**a**) $w_3=0.1$ and (**b**) 0.45. This region is filled with bright red. The blue hatched region is the possible range without the constraint on $w_4$.

The force distribution is modulated owing to the multiple incident ultrasounds. When the frequency difference is perceptible (e.g., $\delta \omega /(2\pi )$ is lower than 1 Hz), we observe the change in the acoustic radiation force of the order of a few seconds. We demonstrate the periodic on/off switching of the acoustic radiation force based on the modulated field. Figure 2a shows a numerical example of time response to illustrate the switching behavior at an interval of 4 s. The strength of the field distribution $F_{\mathrm {z}}(z)$ is weighted according to $F_{\mathrm {t}}(t)$. At approximately $t=0$, 4 s, and 8 s, we have *on state*: $F \approx 2.6F_{\mathrm {z}}(z)$. At $t=2$ s and 6 s, we have *off state*, in which we observe the considerably small force: $F \approx 0$. To realize such switching behavior, we used $N=6$ and the weights $w_0,\ldots ,w_{5}$, as shown in Fig. 2b.

For further understanding, we illustrate the time variation of the force distribution at $t=2$ s, 3.2 s, and 4 s in Fig. 3. The acoustic radiation force is periodically turned on and off at specific intervals. Note that the sign of $F_{\mathrm {z}}$ depends on the acoustophoretic contrast factor $\Phi$, i.e., $F_{\mathrm {z}} \propto + \sin (2kz)$ for $\Phi >0$ and $F_{\mathrm {z}} = - \sin (2kz)$ for $\Phi <0$^24^. In our study, the contrast factor was assumed to be positive.

The time response of the acoustic radiation force is programmable via the weights provided by the multitone field. To understand the tunability, we plotted the possible weight values at $N=4$ on the parallel coordinates as shown in Fig. 4. We introduced the constraint $\sum _{n=1}^{N}\tilde{p}_n^2 =1$ to maintain the acoustic energy density as a constant. Furthermore, we calculated the possible range of $w_1$ and $w_2$ for specific values of $w_3$: (a) $w_3=0.1$ and (b) 0.45. From Eq. (23), we plotted the relation between $w_1$ and $w_2$ as shown in Fig. 5. The blue-hatched region is the possible range without the constraint on $w_3$.

### Experimental demonstration for periodic switching

**Figure 6:**
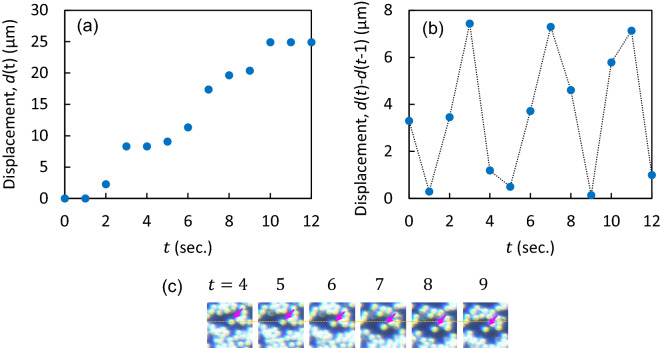
(**a**) Deviation of the position of a certain particle with respect to the reference position at $t=0$. The displacement is measured from the optical microscope images. (**b**) Mean displacement every 1 s for 30 particles. (**c**) Snapshots of the particle motion in (**a**) for several seconds. Orange dashed line indicates the position at $t=4$ s. The particle of interest is indicated by the pink arrow.

We verified the switching behavior through measurements. In the initial setup, the particles were uniformly distributed in the channel. We examined the trajectory of the particles imposed by the synthesized ultrasounds that were periodically switched at an interval of 4 s, see Fig. 2a. Figure 6a shows the deviation in the position of a certain particle with respect to a reference position over time. Moreover, Fig. 6b shows the mean displacement every 1 s for 30 particles. The particles drifted steeply at intervals of approximately four seconds; this drift can be attributed to the periodic drag induced by the restless acoustic radiation force on the particles in the microfluidic channels.

Furthermore, the snapshots of the particle motion from $t=4$ s to 9 s are shown in Fig. 6c. We observed that the particle moved away from the reference plane (orange dotted line) over time with a significantly steep drift between $t=6$ s and 8 s. This can be attributed to the switching of the force applied to the particle.

## Discussion

The acoustic radiation force is typically discussed in the context of a field that oscillates monotonically with the specific angular frequency, $\omega _0$. Then, substituting $N=1$ to Eq. (12), the harmonic series is simply written as the single term, $\varvec{F}(\varvec{r},t)=\varvec{F}_0(\varvec{r})$, due to $\cos (i\delta \omega t)=1$ at $i=0$. This indicates that the acoustic radiation force is independent of time, i.e., it is described as the function of the spatial coordinate, $\varvec{r}$. In contrast, when there are multiple incident fields at $N \ge 2$, the acoustic radiation force is expressed as the sum of harmonics at the fundamental frequency, $\delta \omega$. Furthermore, we observe that the resulting acoustic radiation force is the beat that experiences interference from the time-varying forces with multiple frequencies.

According to Eq. (13), the distribution of the acoustic radiation force varies in time resulting from the harmonics $\cos (i \delta \omega t)$. Thus, the switching period of acoustic radiation force is determined by the frequency difference $\delta \omega$. Specifically, in our analysis, we set the fundamental angular frequency $\delta \omega/(2\pi)$ = 0.25 Hz, i.e., the switching period $2\pi/\delta \omega$ = 4 s.

Interestingly, the acoustic radiation force is not considered as a superposition of the fields $\phi _1,\ldots , \phi _N$ because it has nonlinear behavior owing to the squaring operation ($p^2$ and $v^2$); see Eq. (2). This nonlinear effect leads to the complicated description of the acoustic radiation force in the synthesized field, which is represented as a superposition of the harmonics with the amplitudes $\varvec{F}_0,\ldots ,$ $\varvec{F}_{N-1}$.

From the tunability analysis, we see that the steady component takes the constant value: $w_0=\sum _{n=1}^{N}\tilde{p}_n^2 =1$, see Fig. 4. The acoustic radiation force created by the synthesized ultrasound includes a time-invariant (steady) component that is the sum of the radiation force created by the monotone fields. In contrast, the weight of the higher-order components, $w_1, \ldots ,w_{N-1}$, varies within the specific range.

Moreover, both $w_1$ and $w_2$ have a large range of values (i.e., red region in Fig. 5a) when the value of the higher order weight is small, i.e., $w_3=0.1$. On the contrary, $w_2$ and $w_3$ exist in the narrow region (i.e., red region in Fig. 5b) at $w_3=0.45$. This is because the weight is associated with the multivariable polynomial, as shown in Eq. (23). Therefore a large (small) value of $w_3$ gives rise to the narrow (wide) possible range of $w_1$ and $w_2$. From Eq. (23), we also see that the tunability of the lower order weight is more flexible than that of the higher order weight, since it is described using a larger number of variables. For instance, $w_1$ is the function of $\tilde{p}_1$, $\tilde{p}_2$, $\tilde{p}_3$, and $\tilde{p}_4$ whereas $w_3$ is that of only $\tilde{p}_1$ and $\tilde{p}_4$ at $N=4$.

 In conclusion, we have demonstrated that the beat of the acoustic radiation force arises from the synthesized field. We have theoretically and experimentally shown that the synthesized field allows the periodic on/off switching of the acoustic radiation force for one denominational planar standing wave in the straight microfluidic channel. Our study provides a method to temporally manipulate acoustic radiation force. Thus, our theoretical and experimental investigation opens up the possibility of programmable particle and cell manipulations based on the acoustic radiation force without the active controls. This paper has considered only the quantized relative phase, i.e., $\theta _n-\theta _m\in [0,\pi ]$, of the field. We expect that the fine-tuned amplitudes and relative phases of the fields increase the flexibility of the temporal manipulation of the acoustic radiation force.

## Methods

**Figure 7:**
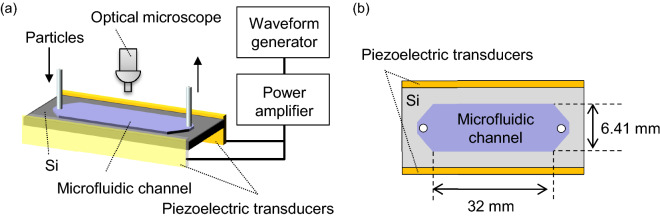
(**a**) Overview of measurement setup. (**b**) Top view of the channel.

Figure 7a,b illustrate the measurement setup for the microfluidic channel. Our microfluidic channels (depth = 180 $\upmu$m, width = 6.41 mm, and length = 32 mm) were manufactured using the standard semiconductor fabrication process. A silicon wafer of 500 $\upmu$m was etched by the deep reactive ion etching. Following this, the top-side of the silicon wafer was sealed with a glass plate of 700 $\upmu$m thickness via anodic bonding. The wafer was subsequently diced into small chips, the glass plate was drilled, and the silicon tubes were glued to both edges of the channel. In the next step, piezoelectric transducers (HC-2015S12, Honda Electronics Co., Ltd.) were attached to both the longer sides of the chip, and water containing the small particles was filled in the channel using a piezoelectric micropump (SDMP302, Takasago Fluidic Systems). We used particles of diameter 10.57 $\upmu$m with 0.11 $\mu$m standard deviation (PS-ST-10.6, Microparticles GmbH) at a concentration of 0.05% w/v.

To mimic the ultrasounds emitted from the multiple sources, we applied the synthesized multitone voltage $\sum _{n=1}^{6}V_n\sin (\omega _nt+\eta _n)$ to the piezoelectric elements using an arbitrary waveform generator (Mi2.6021, SPECTRUM) with control software (SBench6-Pro 6.1). Thus, the synthesized field was induced in the microfluidic chip. The driving frequency was $\omega _n = \omega _0+(n-1)\delta \omega$ with $\omega _0/(2\pi )=117$ kHz and $\delta \omega /(2\pi )=$ 0.25 Hz. The power of the waveform generator was amplified with a power amplifier (HSA 2011, NF Corp.). The peak-to-peak voltage at the amplifier output was 40 V. The trajectory of the particles was recorded using an optical microscope (SMZ-10, Nikon Corp.) equipped with a CMOS camera (WRAYCAM-NOA630, WRAYMER Inc.) at a capture rate of 1 Hz. It must also be noted that the channel width was approximately matched to the half-wavelength to create a one-dimensional resonant standing wave.
